# Toward the development of a sporadic model of Alzheimer's disease: comparing pathologies between humanized APP and the familial J20 mouse models

**DOI:** 10.3389/fnagi.2024.1421900

**Published:** 2024-07-08

**Authors:** Peggy Rentsch, Kiruthika Ganesan, Alexander Langdon, Lyndsey M. Konen, Bryce Vissel

**Affiliations:** ^1^Centre for Neuroscience and Regenerative Medicine, St. Vincent's Centre for Applied Medical Research, St Vincent's Hospital, Sydney, NSW, Australia; ^2^UNSW St Vincent's Clinical School, Faculty of Medicine, University of New South Wales, Sydney, NSW, Australia; ^3^School of Life Sciences, Faculty of Science, University of Technology Sydney, Sydney, NSW, Australia

**Keywords:** humanized Aβ, J20, sporadic and familial Alzheimer's disease, neuroinflammation, lipopolysaccharide, dendritic spine density

## Abstract

**Background:**

Finding successful therapies for individuals with Alzheimer's disease (AD) remains an ongoing challenge. One contributing factor is that the mouse models commonly used in preclinical research primarily mimic the familial form of AD, whereas the vast majority of human cases are sporadic. Accordingly, for a sporadic mouse model of AD, incorporating the multifactorial aspects of the disease is of utmost importance.

**Methods:**

In the current study, we exposed humanized Aβ knock-in mice (hAβ-KI) to weekly low-dose lipopolysaccharide (LPS) injections until 24 weeks of age and compared the development of AD pathologies to the familial AD mouse model known as the J20 mice.

**Results:**

At the early time point of 24 weeks, hAβ-KI mice and J20 mice exhibited spatial memory impairments in the Barnes maze. Strikingly, both hAβ-KI mice and J20 mice showed significant loss of dendritic spines when compared to WT controls, despite the absence of Aβ plaques in hAβ-KI mice at 24 weeks of age. Glial cell numbers remained unchanged in hAβ-KI mice compared to WT, and LPS exposure in hAβ-KI mice did not result in memory deficits and failed to exacerbate any other examined AD pathology.

**Conclusion:**

The study highlights the potential of hAβ-KI mice as a model for sporadic AD, demonstrating early cognitive deficits and synaptic alterations despite no evidence of Aβ plaque formation. These findings underscore the importance of considering multifactorial influences in sporadic AD pathogenesis and the need for innovative models to advance our understanding and treatment strategies for this complex disease.

## Introduction

Although decades of research have been invested in finding a treatment for Alzheimer's disease (AD), there remains no cure for patients. One possible reason for these failures is that putative therapeutics are screened in animal models based on familial AD (FAD), yet only 1%−5% of patients fall in this category (Bekris et al., 2010; Hall and Roberson, 2012; Kitazawa et al., 2012; Dorszewska et al., 2016). The remaining 95%−99% of patients suffer from sporadic late-onset AD (LOAD), characterized by a complex and multifactorial etiology, and, as a result, no mouse models faithfully represent these cases to date (Beydoun et al., 2014; Chakrabarti et al., 2015; Morris et al., 2018; Foidl and Humpel, 2020). Accordingly, there is a critical need to develop novel sporadic AD mouse models that more accurately resemble the wide range of AD pathologies found in humans.

Although Aβ is a well-established hallmark of AD, the mechanisms through which it is produced and contributes to the overall AD pathology are likely to be different between familial and sporadic AD. Put simply, in familial AD and its animal models, symptoms are caused by Aβ's over-production as a result of DNA mutations to APP or related proteins (Hall and Roberson, 2012). In sporadic AD, however, the causes of Aβ production are unclear (Huang and Mucke, 2012; Barykin et al., 2017). To better understand this, a mouse model with only a humanized APP gene was recently created, known as the hAβ-knock in (hAβ-KI) (Baglietto-Vargas et al., 2021). This means that like familial AD models, hAβ-KI mice can produce human Aβ peptides from APP. These human peptides are known to aggregate more readily and are more toxic than murine Aβ (Lv et al., 2013). Unlike familial AD models, however, hAβ-KI mice do not overexpress Aβ and are therefore expected to produce human Aβ at physiological levels in the absence of other familial DNA mutations (Baglietto-Vargas et al., 2021). Recent research utilizing these mice has shown the development of cognitive deficits, synaptic alterations and neuroinflammation with age, therefore leading the way to the development of a sporadic AD mouse model (Baglietto-Vargas et al., 2021; Kshirsagar et al., 2022).

While Aβ is a component, it is unlikely the single cause of sporadic AD. In fact, the influence of various risk factors are believed to predispose an individual toward developing AD including proinflammatory events leading to neuroinflammation (Breunig et al., 2013; Fann et al., 2018; Kempuraj et al., 2020; Leng et al., 2022; Thakur et al., 2023), risk genes (Harold et al., 2009; Lambert et al., 2009; Wightman et al., 2021; Chung et al., 2023), diet (Solfrizzi et al., 2011; Baranowski et al., 2020), metal exposure (Bakulski et al., 2020), and comorbidities such as vascular disease (Cortes-Canteli and Iadecola, 2020), diabetes (Arvanitakis et al., 2004) and depression (Ownby et al., 2006). Accordingly, to model the multifactorial risks associated with sporadic AD and to potentially accelerate the development of an AD phenotype in mice, hAβ-KI mice should be exposed to these risk factors.

Toward this goal, in the current study, adult hAβ-KI mice received weekly low-dose LPS injections (0.2 mg/kg) until 24 weeks of age to model chronic low-grade inflammation that can occur during life such as a recurring infection, head injury, stress, or biological dysfunction. By comparing the putative sporadic AD hAβ-KI mice to the well-established familial AD J20 mouse model, we confirmed memory impairments in the J20 mice and established that 6 month old hAβ-KI mice also display early spatial memory deficits. Strikingly, we further uncovered a comparable decrease in spine density between J20 and hAβ-KI mice when compared to WT controls. These AD pathologies were present in hAβ-KI mice despite the absence of Aβ plaques, while J20 mice showed abundant plaque deposition at this age as previously reported (Wright et al., 2013). Lastly, glial cell populations within the hippocampus remained unchanged in hAβ-KI mice compared to WTs and LPS did not worsen any AD pathologies.

## Methods

### Animals

The humanized *App* knock-in strain used in this study was originally obtained from the Jackson Laboratory (strain # 031050). The hAβ-KI mouse line (B6J(Cg)-*App*^*tm*1.1*Aduci*^/J) expresses humanized App through the introduction of 3 point mutations into the Aβ coding sequence of exon 14 of the mouse *App* gene. Additionally, these mice also possess loxP sequences flanking exon 14. Only homozygous mice of both sexes were utilized in this study. Hemizygous transgenic J20 [also known as hAPP-J20 (Wright et al., 2013)] and non-transgenic wildtype (WT) littermates from the B6.Cg-Tg (PDGFB-APPSwInd) 20Lms/2J (J20; JAX strain # 034836) line of both sexes were utilized. J20 mice overexpress mutant APP (amyloid precursor protein) harboring both the Swedish and Indiana mutations and regulated by the PDGF-β chain promoter. All mice were bred on a C57BL/6J background. All mice were bred and maintained at Australian BioResources (Moss Vale, Australia) until experimentation. During experiments, mice were kept in groups of up to five per cage and maintained on a 12-h light/dark cycle with access to food and water *ad libitum*. Animal experiments were performed with the approval of the Garvan Institute and St. Vincent's Hospital Animal Ethics Committee under approval number 17/28 and in accordance with the Australian National Health and Medical Research Council animal experimentation guidelines and the local Code of Practice for the Care and Use of Animals for Scientific Purposes.

### Lipopolysaccharide injections and experimental timeline

The hAβ-KI mice received intraperitoneal injections of low dose (0.2 mg/kg in Saline) Lipopolysaccharide (LPS, *Escherichia coli* O111:B4, L3024, Sigma Aldrich) or saline weekly for 17 weeks (i.e. starting at 8 weeks of age until 24 weeks). One day following the last injection, behavioral testing commenced with the Open Field (OFT) and Barnes Maze test. The acquisition trial for Barnes Maze was performed over 5 days followed by a probe trial on the 6th day. Mice were sacrificed the day after the probe trial.

### Behavior tests

#### Open field test

The OFT chambers measured 273 mm × 273 mm with 203 mm high glass walls and were placed inside a sound-attenuating cubicle (MED-OFAS-MSU, MED-OFA-022, Med Associates inc.). Mice were introduced into the central area of the chamber and given 10 min to explore the open field environment. The movement of the animals was recorded using Activity Monitor 7 (Med Associates Inc.), which employs infrared beams for activity detection within the chamber. The distance traveled by each animal over the 10-min period was measured to evaluate locomotor activity, while the duration spent by the animal in the central area of the arena served as an indicator of anxiety.

#### Barnes maze test

The Barnes Maze setup comprised a circular platform measuring 920 mm in diameter, situated 1 m above ground. Around the platform's perimeter, 20 holes of 50 mm diameter were uniformly distributed. Beneath one of the holes, a concealed black escape box [dimensions: 175 mm (D) × 75 mm (W) × 80 mm (H)] was positioned, while the remaining holes were blocked. Animal movements were monitored using the ANYmaze Video Tracking System 6.33 (Stoelting Co.) with a camera (DMK 22AUC03) directly positioned above the maze. We followed the Barnes maze protocol described previously (Ganesan et al., 2024). In short, during the acquisition phase (three trials per day for 5 days), mice were given 2 min to locate the hidden escape box, or manually guided to the escape box after the 2 min elapsed. The escape box location was randomized for each mouse but consistent throughout the trials. For acquisition phase the average of the three trials per day was used for analysis. The day after the last acquisition trial, a single 90 s probe trial was performed, where memory retention was tested without the escape box present. Performance metrics included primary latency, path length and errors as well as time spent in each quadrant. The investigator performing the experiment was blinded for genotype and treatment.

### Golgi staining

Golgi staining was utilized to visualize dendritic spines using the FD Rapid GolgiStain Kit (PK401, FD NeuroTechnologies, inc.) according to the manufacturer's instructions. Briefly, brains of anesthetized mice were extracted, impregnated in a solution (prepared by mixing equal parts of solution A and solution B from the kit) and stored in darkness for 2 weeks. Brains were changed to solution C and stored in darkness for 3 days. On the final day, brains were snap-frozen [with dry ice and isopentane (Sigma Aldrich, M32631)], 100 μm thick coronal sections were cut using a cryostat, sections were slide mounted onto gelatin-coated slides (1% Gelatin, Sigma Aldrich, G9391; 0.1% Chromium potassium sulfate dodecahydrate, Sigma Aldrich, 243361) and left to dry overnight. On the next day, staining was performed according to the manufacturer's protocol and slides were cover-slipped with Permount mounting media (Thermo FischerScientific, SP15).

### Immunostaining

Mice were anesthetized with ketamine (8.7 mg/ml) and xylazine (2 mg/ml), transcardially perfused with 4% paraformaldehyde (PFA) followed by post-fixing in 4% PFA overnight and stored in 30% sucrose. Brain sections of 40 μm thickness were cut using a cryostat. Free-floating sections were washed with PBS three times and blocked with 3% BSA (Bovogen Biologicals, BSAS 1.0) + 0.25% Triton (Sigma Aldrich, T8787) in 1 × PBS for an hour at room temperature. After blocking, the sections were incubated in the following primary antibodies for 72 h at 4°C: rabbit polyclonal IBA1 (Labome, Wako Chemicals USA, 019-19741), rabbit polyclonal GFAP (Dako Z0334). All sections were washed three times with PBS and incubated in the secondary antibody, donkey anti-rabbit 488 (Invitrogen, A32790). Subsequently, sections were washed with PBS three times and counterstained with DAPI (Invitrogen, D1306) for 10 min at room temperature. Finally, the sections were mounted onto SuperFrost slides (ThermoFisher Scientific, SuperFrost plus F41296SP) and coverslipped (Menzel-Glasser, #1) with 50% glycerol mounting medium (Sigma Aldrich, GG1). For detection of Aβ plaques, sections were incubated in a biotinylated 6E10 antibody (1:1,000, Covance) for 24 h at 4°C and subjected to HRP-labeled avidin-biotin complex and DAB.

### Aβ plaque quantification

Quantification of Aβ plaques was conducted in the hippocampal region subfield from five sections per brain (Bregma −1.34 to −2.30 mm). Slides were imaged using an Axio Imager M2 upright brightfield microscope at 10 × magnification (Carl Zeiss Pty Ltd). All plaque counts were conducted manually and the investigator was blind to genotype and treatment.

### Analysis

#### Neurolucida

Spine analysis was performed using Neurolucida (MBF Biosciences). For each brain, three secondary dendrites (both apical and basal), of branch orders 2–8 were chosen from four random neurons of the dorsal hippocampal CA1 region (Bregma −1.58 to −2.30 mm based on Paxinos atlas for mouse brain). Selected dendrites were traced at 100 × magnification (Axio Imager M2), and spines were counted manually. The tracings were exported to Neurolucida Explorer to perform spine analysis. Spine density was represented as the number of spines per 10 μm length of the dendrite. The Investigator was blinded for genotype and treatment.

#### Stereology

Glial cells were counted in the dorsal hippocampus (Bregma −1.34 to −2.30 mm based on Paxinos atlas for mouse brain) using the Optical Fractionator module in Stereo Investigator (MBF Biosciences). Every sixth section was quantified with a total of five sections per brain. Using an Axio Imager Z2 fluorescent microscope, the region of interest was traced at a low 5 × magnification and cells were quantified at 40 × using a counting frame of 100 μm × 100 μm and a grid size of 200 μm × 200 μm. The guard zone height was set as 5 μm and the dissector height was set to 10 μm for all sections. To exclude the differences in traced volume, cell counts were represented as number of cells per volume. The Investigator was blinded for genotype and treatment.

### Statistics

All statistical analyses were performed using Graphpad Prism 8.4.3. Differences between mean were assessed using either a one-way, two-way ANOVA or two-way ANOVA with repeated measures, followed by *post-hoc* Bonferroni analysis where applicable. For all analyses, a *p*-value of ≤ 0.05 was considered significant.

## Results

**Figure 1:**
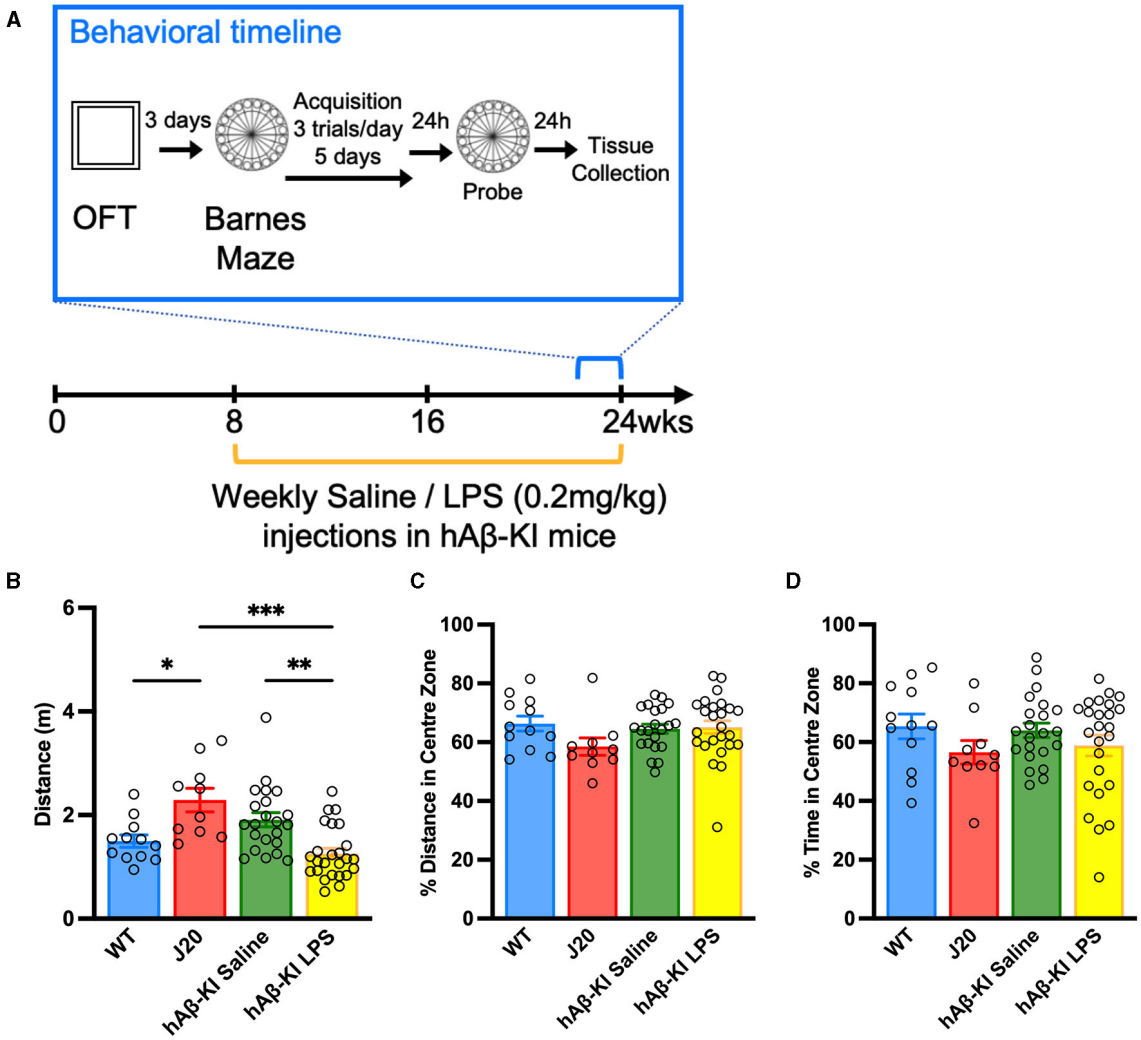
Repeated LPS administration reduces locomotor behavior in hAβ-KI mice. **(A)** Experimental timeline. In the Open field test **(B)** J20 mice traveled a greater distance compared to WT mice and LPS reduced the distance traveled in hAβ-KI mice compared to saline in injected controls, while **(C)** the percentage of distance and **(D)** time spent in the center zone was unchanged between groups, indicating no changes in anxiety. All values represent the mean ± standard error of the mean (SEM). WT *n* = 12, J20 *n* = 10, hAβ-KI saline *n* = 22, hAβ-KI LPS *n* = 25. *p < 0.05, **p < 0.01, ***p < 0.001.

We set out to generate a sporadic model of AD for pre-clinical research and compare it to the well-established familial AD J20 mouse line. To achieve this, humanized hAβ-KI mice were given weekly low-dose LPS injections of 0.2 mg/kg, starting at 8 weeks of age until 24 weeks to model chronic inflammation. At 24 weeks of age, all mice underwent behavioral tests assessing general motor behavior and memory deficits, after which tissue was collected for spine analysis, Aβ plaque and glial cell quantifications (Figure 1A). In the following results, data from both genders are presented together, as significant results or trends were not obtained by one gender exclusively.

### LPS induced hypoactivity in humanized hAβ-KI in the open field test

Given the prolonged administration of LPS previously associated with impaired motor output (Kozak et al., 1994; Lasselin et al., 2020), it was crucial to initially assess general behavioral alterations in humanized hAβ-KI mice before conducting memory tests. Accordingly, WT, J20 and hAβ-KI (injected weekly with either saline or 0.2 mg/kg LPS) were checked for abnormalities in locomotor and anxiety-like behavior in an Open Field Test (OFT). A one-way ANOVA revealed a significant effect in the total distance traveled between groups [*F*_(3, 65)_ = 9.914, *p* < 0.001; Figure 1B]. Bonferroni *post-hoc* analysis indicated that J20 mice covered a greater distance compared to WT mice (*p* < 0.05) and hAβ-KI mice injected with LPS (*p* < 0.001). Interestingly, LPS administration in hAβ-KI mice led to reduced distance traveled compared to those injected with saline (*p* < 0.01). Additionally, the percentage of distance and time spent in the center zone of the arena, an indicator of anxiety in the open field test, was measured. The one-way ANOVA analyses did not show significance, indicating no variations in anxiety levels across the animal groups [Distance: *F*_(3, 65)_ = 1.532, *p* = 0.21; Figure 1C, Time: *F*_(3, 65)_ = 1.132, *p* = 0.3428; Figure 1D]. Combined, these results indicate that J20 mice exhibit significantly increased levels of locomotor activity and, conversely, LPS reduced motor activity in hAβ-KI mice.

### Spatial memory is impaired in hAβ-KI mice

**Figure 2:**
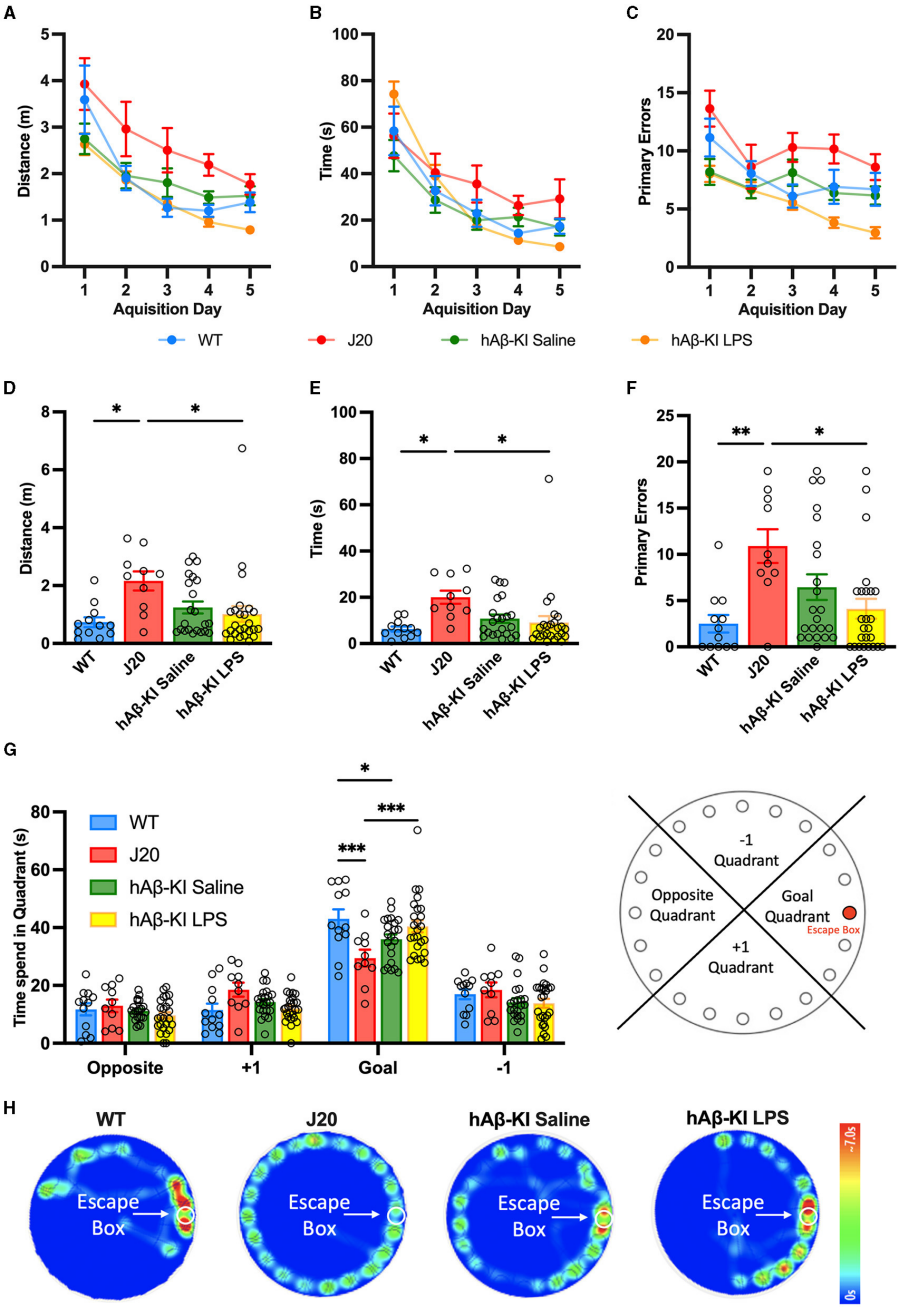
hAβ-KI mice develop early spatial memory deficits in the Barnes maze. During the acquisition phase, all experimental groups learned the location of the escape box over time measured as a reduction in **(A)** distance traveled, **(B)** primary latency and **(C)** primary errors made until reaching the escape box. During the probe trial, J20 mice **(D)** traveled a greater distance, **(E)** took longer and **(F)** made more primary errors in finding the escape box compared to WT mice and hAβ-KI mice injected with LPS. **(G)** J20 and hAβ-KI mice spent significantly less time in the goal quadrant on the probe trial day compared to WT mice, indicating memory deficits. **(H)** Heat map showing animal's activity during the probe trial. All values represent the mean ± standard error of the mean (SEM). WT *n* = 12, J20 *n* = 10, hAβ-KI saline *n* = 22, hAβ-KI LPS *n* = 25. *p < 0.05, **p < 0.01, ***p < 0.001.

After having observed that J20 and hAβ-KI mice treated with LPS showed slight hyper- and hypoactivity in the OFT respectively, we proceeded to assess memory deficits in these mice using the Barnes Maze Test. In this test, animals are trained to associate spatial cues with the location of an escape box during the acquisition phase and remember its location even in the absence of the escape box during the probe trial. Accordingly, the Barnes Maze assesses the animal's ability to learn, retain and retrieve spatial memory. During the acquisition phase, we measured primary distance, latency and errors to locate the escape box. If the hyper- and hypoactivity of J20 and hAβ-KI mice treated with LPS did not significantly alter the animal's exploratory behavior, it would be expected that over time all animals learn the location of the escape box similarly. The primary distance traveled to locate the escape box was measured and a two-way repeated measures ANOVA revealed a significant effect of trial day [*F*_(2.558, 166.3)_ = 28.69, *p* < 0.001] and experimental group [*F*_(3, 65)_ = 10.08, *p* < 0.001] with no significant interaction [*F*_(12, 260)_ = 0.9016, *p* = 0.5459; Figure 2A]. A Bonferroni multiple comparisons test revealed a significant decrease in the distance traveled by all groups in the first 3 days (*p* < 0.05) after which all groups traveled a similar distance to reach the escape box. Furthermore, by day 5 there was no significant difference in distance traveled between experimental groups. This indicates that by day 5 the memory of the escape box location was consistent between all groups. Next, a two-way repeated measures ANOVA compared the primary latency over acquisition trial days for all experimental groups. There was a significant interaction between the trial days and experimental groups [*F*_(12, 260)_ = 0.3494, *p* < 0.001; Figure 2B] as well as a significant effect of the trial days [*F*_(2.381, 154.7)_ = 53.00, *p* < 0.001] but not the experimental groups [*F*_(3, 65)_ = 1.483, *p* = 0.2273] on the latency. Bonferroni *post-hoc* analysis revealed that by day 5 there were no statistical differences between trial day in any of the experimental groups and no differences between the experimental groups, indicating that all groups learned the task equally. Lastly, primary errors were measured and a two-way repeated measures ANOVA revealed a significant effect of trial day [*F*_(3.508, 228.0)_ = 9.693, *p* < 0.001] and experimental group [*F*_(3, 65)_ = 16.5, *p* < 0.001] with no significant interaction [*F*_(12, 260)_ = 0.1269, *p* = 0.2370; Figure 2C]. A Bonferroni multiple comparisons test revealed that by day 5 all groups stagnated in the number of errors made to find the escape box as there were no significant differences between day 4 and 5 for any group. However, even on day 5 hAβ-KI mice treated with LPS made significantly fewer errors when compared to WT (*p* < 0.05), J20 (*p* < 0.001), and hAβ-KI mice treated with saline (*p* < 0.01), possibly indicating a better memory of the escape box location.

On the probe test day, we first assessed the primary measures at which experimental groups searched for the former location of the escape box and a one-way ANOVA found significant differences amongst the groups for distance [*F*_(3, 65)_ = 3.564, *p* < 0.05; Figure 2D], latency [*F*_(3, 65)_ = 3.652, *p* < 0.05; Figure 2E] and errors [*F*_(3, 65)_ = 5.084, *p* < 0.01; Figure 2F]. Bonferroni *post-hoc* analysis revealed that J20 mice traveled a significantly longer distance (*p* < 0.05), taking more time (*p* < 0.05) and making more errors (*p* < 0.05 compared to WT and *p* < 0.01 compared to hAβ-KI mice treated with LPS) to find the escape box location when compared to WT and hAβ-KI mice treated with LPS. In addition to these primary measures, we next assessed the total time spent in each goal quadrant during the duration of the probe trial, a measure previously indicated to be more sensitive as it is not influenced by chance of starting position, anxiety, motivation, and exploratory behavior (O'Leary and Brown, 2013). A two-way ANOVA revealed a significant interaction between different quadrants and experimental groups [*F*_(9, 260)_ = 3.85, *p* < 0.001; Figures 2G, H], as well as a significant effect of quadrant [*F*_(3, 260)_ = 162, *p* < 0.001] but not the experimental groups [*F*_(3, 260)_ = 0.901, *p* = 0.44]. A Bonferroni *post-hoc* analysis confirmed that J20 mice (29.463 ± 2.874) spent significantly less time in the goal quadrant when compared to WT (43.016 ± 3.29; *p* < 0.001) and hAβ-KI mice treated with LPS (40.46 ± 2.074;*p* < 0.001). Strikingly, we also revealed a significant reduction of time spent in the goal quadrant of hAβ-KI mice treated with saline (35.977 ± 1.623) when compared to WT mice (*p* < 0.05). Collectively, we detected memory deficits in both J20 mice and hAβ-KI mice treated with saline, a critical feature to model sporadic AD. Interestingly, exposing hAβ-KI mice to LPS abolished the memory deficit observed in hAβ-KI mice, indicating that LPS did not worsen this AD phenotype.

### Dendritic spine loss in hAβ-KI is equivalent to that seen in J20 mice

**Figure 3:**
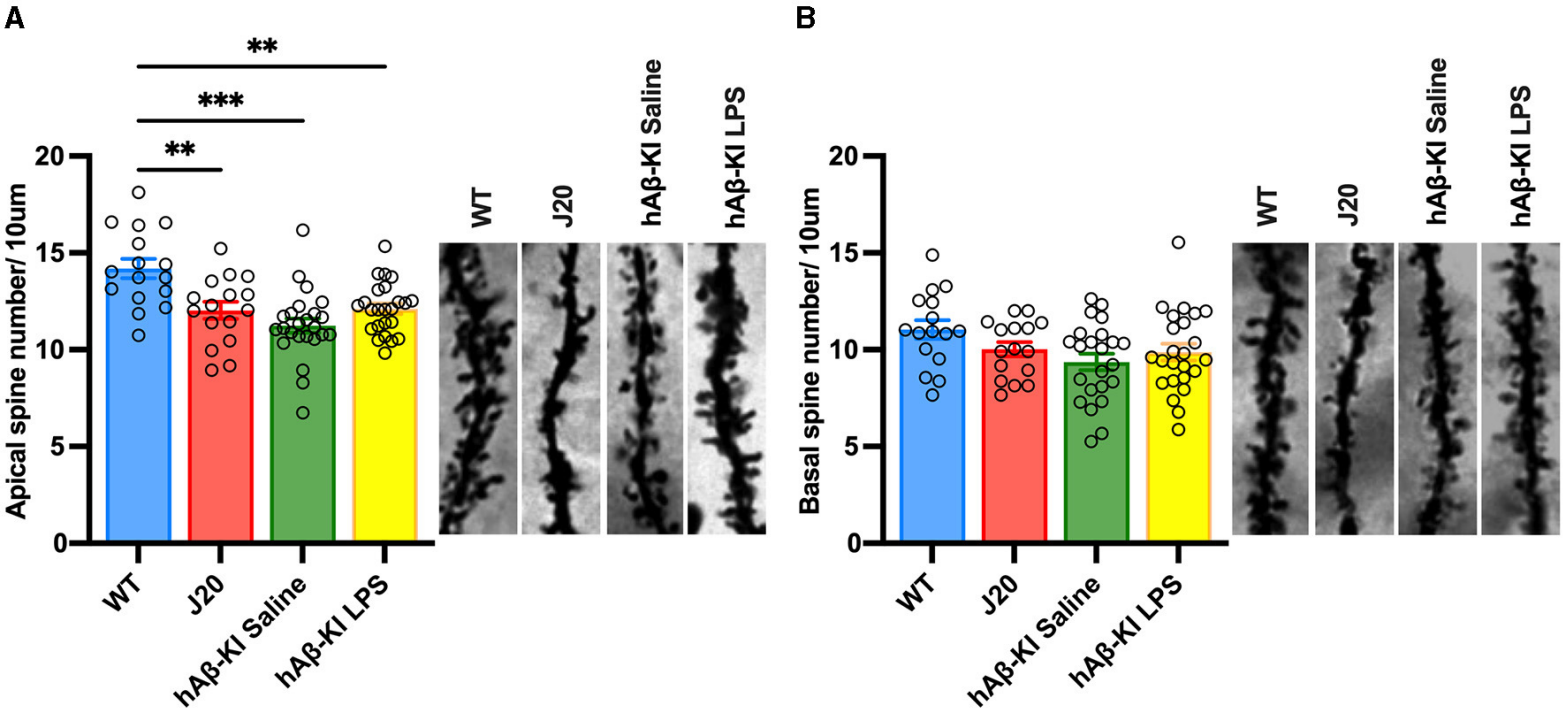
hAβ-KI mice have equivalent apical dendritic spine loss to J20 mice when compared to WT mice. Quantification of spine density revealed a significant reduction of spines in J20 mice and hAβ-KI mice injected with either LPS or saline compared to WT mice in **(A)** apical but not **(B)** basal dendrites. All values represent the mean ± standard error of the mean (SEM). *n* = 2 dendritic branches/neuron with two neurons/brain and *n* = 4–6 brains/group (WT *n* = 4, J20 *n* = 4, hAβ-KI saline *n* = 6, hAβ-KI LPS *n* = 6). **p < 0.01, ***p < 0.001.

In addition to memory deficits, hippocampal synapse loss and neurodegeneration are major hallmarks in the human AD brain (Brun and Englund, 1981; Terry et al., 1991; De Wilde et al., 2016; Mecca et al., 2022). Hence, a putative sporadic AD mouse model needs to resemble this phenotype. We and others have previously shown the familial J20 mouse model of AD mirrors neuronal and synaptic degeneration in the hippocampus (Wright et al., 2013, 2023; Hong et al., 2016). In the present study, spine quantification using Golgi-impregnated tissue corroborated our previous result, with a one-way ANOVA confirming significant spine loss in apical [*F*_(3, 76)_ = 9.83, *p* < 0.001; Figure 3A] but not basal [*F*_(3, 76)_ = 2.381, *p* = 0.0762; Figure 3B] dendrites between groups. Strikingly, Bonferroni *post-hoc* analysis revealed that hAβ-KI mice [treated with either saline (*p* < 0.001) or LPS (*p* < 0.01)] displayed similar amounts of spine loss to J20 mice (*p* < 0.01) when compared to WT mice. Collectively, the concurrent appearance of significant spine pathology with the development of memory deficits in hAβ-KI mice is a crucial step toward a reliable model of sporadic AD.

### No changes to glial populations in the hippocampus of hAβ-KI mice

**Figure 4:**
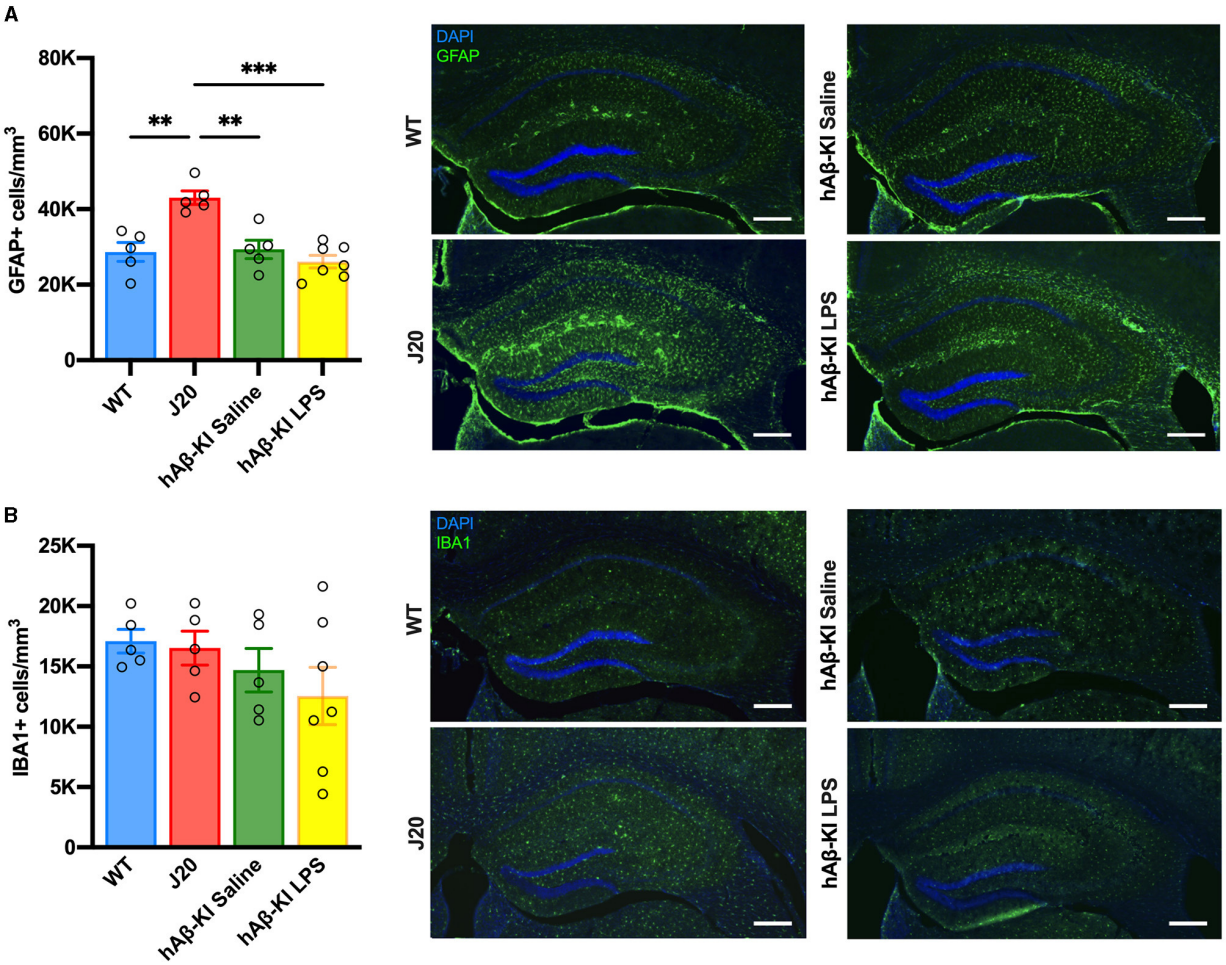
J20 mice have increased GFAP^+^ astrocytes, with no changes in IBA1^+^ microglia between groups in the hippocampus. Stereological quantification in the dorsal hippocampus revealed **(A)** an increase in GFAP^+^ astrocytes when compared to any other group and **(B)** no changes between groups in IBA1^+^ microglial cells. Scale bar 400 μm. All values represent the mean ± standard error of the mean (SEM). WT *n* = 5, J20 *n* = 5, hAβ-KI saline *n* = 5, hAβ-KI LPS *n* = 7. **p < 0.01, ***p < 0.001.

Another key feature of AD pathology is the migration, proliferation and activation of glial cells (Breunig et al., 2013; Fann et al., 2018; Kempuraj et al., 2020; Leng et al., 2022; Thakur et al., 2023). We have previously reported changes in glial cell activation in J20 mice (Wright et al., 2013). Thus, we aimed to compare changes in microglia and astrocyte cell populations between J20 and hAβ-KI mice in the current study. Quantification of microglia (represented as IBA1^+^ cells) and astrocytes (represented as GFAP^+^ cells) in the hippocampus using stereology revealed a significant difference only for astrocyte [*F*_(3, 18)_ = 13.2, *p* < 0.001; Figure 4A] but not microglia [*F*_(3, 18)_ = 1.27, *p* = 0.31; Figure 4B] cell populations. Bonferroni *post-hoc* analysis confirmed an increase in the number of astrocytes in J20 mice when compared to WT (*p* < 0.01) and hAβ-KI mice treated with either saline (*p* < 0.01) or LPS (*p* < 0.001). This suggests that total cell populations of microglia and astrocytes remain unchanged in hAβ-KI mice despite chronic exposure of LPS.

### hAβ-KI mice do not show Aβ plaque pathology at 24 weeks

**Figure 5:**
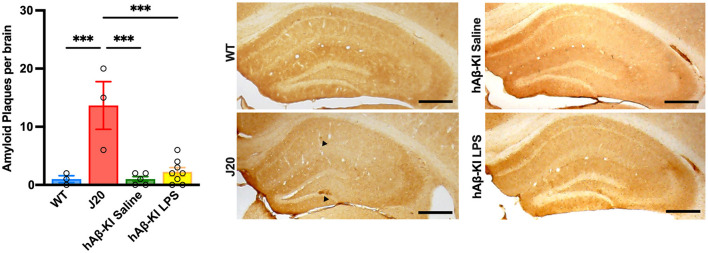
J20 mice display plaque deposition in the hippocampus. 6E10 immunohistochemistry illustrated increased Aβ deposition in J20 mice when compared to any other group. Scale bar 250 μm. All values represent the mean ± standard error of the mean (SEM). WT *n* = 3, J20 *n* = 3, hAβ-KI saline *n* = 5, hAβ-KI LPS *n* = 8. ***p < 0.001.

An important, yet highly debated feature of human AD is the accumulation of the protein Aβ, resulting in plaque formation throughout the brain. In the current study, we used the human-specific antibody 6E10 to measure Aβ deposition in the hippocampus. Quantification of 6E10 immunoreactivity revealed a significant increase in total Aβ plaques between groups [One-way ANOVA *F*_(3, 15)_ = 13.68, *p* < 0.001; Figure 5] and Bonferroni *post-hoc* analysis indicated that exclusively J20 mice had significantly increased Aβ deposition when compared to every other group (*p* < 0.001). Notably, despite finding significant memory deficits and spine loss in hAβ-KI mice at 24 weeks of age, there was little to no Aβ deposition seen at this timepoint.

## Discussion

To combat failing drug developments for AD, comprehensive and accurate sporadic AD mouse models are needed to test putative therapeutics. Accordingly, our study aimed to advance the knowledge of a sporadic AD mouse model, by exposing hAβ-KI mice to low-dose LPS to model chronic inflammation, one of the most prominent environmental risk factors of AD. Here, we are the first to report that hAβ-KI mice develop cognitive and dendritic spine loss as early as 6 months of age, although LPS did not lead to a worsening of the AD pathologies tested.

### hAβ-KI mice develop memory deficits at 6 months of age

Memory deficits are a key feature of AD, and a successful mouse model of sporadic AD needs to recapitulate this phenotype for their utility in research and therapeutic development. To evaluate spatial memory in our study, we utilized the Barnes maze. This test was chosen as the J20 mice are known to have a propensity to float and rely more on thigmotaxis (Karl et al., 2012) in the widely used Morris water maze (Vorhees and Williams, 2014) and the radial arm maze is food-motivated and thus affected by the appetite-suppressing effects of LPS which makes it unsuitable for the present study (Hodges, 1996; Lasselin et al., 2020). Using the Barnes maze, we confirmed memory deficits previously reported via various forms of memory tests (Galvan et al., 2006; deIpolyi et al., 2008; Harris et al., 2010; Karl et al., 2012; Pozueta et al., 2013; Wright et al., 2013) in J20 mice, as during the probe trial J20 mice traveled a longer distance, taking more time and with a higher error rate to find the escape box location compared to WT mice. Although the hAβ-KI mice did not demonstrate a diminished performance in these primary measures, they did however show a deficit when the total time spent in each goal quadrant was assessed during the probe trial. Previous research has indicated that the average time spent in the goal quadrant on the probe trial day is a more sensitive measure of memory compared to the traditional primary measures, as primary measures can be influenced by chance of starting position, anxiety, motivation, and exploratory behavior (O'Leary and Brown, 2013). Accordingly, with this sensitive measure, our study is the first to show spatial memory deficits in hAβ-KI mice as early as 6 months of age. Previous research has shown that this mouse strain shows spatial memory deficits assessed in the Morris water maze at 7 months (Kshirsagar et al., 2022), as well as impaired fear memory at 10 months and recognition memory at 14 months of age (Baglietto-Vargas et al., 2021).

Notably, in our study, the exposure to LPS in hAβ-KI mice did not lead to any memory impairments in any of our measures. It is possible that low-dose chronic LPS exposure led to “immune training” or “LPS training,” a concept by which microglia are preconditioned over time, transforming them toward a neuroprotective phenotype, which in turn could lead to improved behavioral outcomes (Mizobuchi and Soma, 2021). However, LPS is also known to induce depressive behavior alterations, known as “sickness behaviors,” which decrease locomotor activity (Kozak et al., 1994; Lasselin et al., 2020). To avoid these side effects, we intentionally injected only low doses of LPS (0.2 mg/kg), since previous research suggested doses in excess of 0.6 mg/kg induce sickness behaviors (Biesmans et al., 2013; Lasselin et al., 2020). Nevertheless, our OFT data confirmed that LPS did indeed attenuate exploratory behavior in LPS-injected hAβ-KI mice relative to saline-injected controls. This could potentially be attributed to the cumulative effect of weekly LPS injections. Consequently, the absence of a behavioral effect in our LPS-injected hAβ-KI mice in the Barnes maze could be attributed to reduced exploratory behavior. Lastly, future studies could utilize a more challenging Barnes maze protocol, in which the amount of acquisition trials is reduced from 15 to 5 to increase difficulty (Attar et al., 2013). Using this shortened protocol, previous research was able to uncover memory deficits in 4-month-old triple transgenic (3 × Tg) mice that weren't observable using the standard (15 acquisition trials) protocol (Attar et al., 2013).

### hAβ-KI mice have comparable spine loss to J20 mice at 6 months of age

Synapse loss occurs in the early (asymptomatic) stages of AD in patients (Terry et al., 1991; De Wilde et al., 2016; Mecca et al., 2022), is recapitulated in some AD mouse models (Jacobsen et al., 2006; Hong et al., 2016) and remains the best correlate for cognitive deficits. Here, we show that hAβ-KI mice have comparable spine loss in apical dendrites of the CA1 to that of J20 mice when compared to WT counterparts. We are the first to detect this spine pathology as early as 6 months of age in hAβ-KI mice, while previous studies found spine loss and a reduction of synaptic puncta density at the presynaptic level at 7 (Kshirsagar et al., 2022) and 18 months (Baglietto-Vargas et al., 2021), respectively. Future studies could advance the data by correlating synaptic plasticity genes involved in actin polymerization (Arc, BDNF) to spine loss data, to gain molecular insight. The concurrent occurrence of spine loss and cognitive deficits in these mice emphasizes their early and interconnected role in the progression of sporadic AD pathology, further solidifying the causal relationship between these factors.

Although the precise mechanisms leading to synapse loss and cognitive deficits in AD remain unclear, traditionally Aβ plaques have been described as a causal hallmark of the disease. Here, we show that hAβ-KI mice do not develop Aβ plaques at 6 months of age and is in line with others suggesting that this mouse line doesn't develop plaques even at 22 months (Baglietto-Vargas et al., 2021) of age. Although not investigated in our study, Baglietto-Vargas et al. found an increase in insoluble Aβ (including Aβ40 and Aβ42), although the Aβ42/40 ratio remained unchanged. The authors suggested that additional factors are required to trigger Aβ plaque formation, which could be introduced by exposing hAβ-KI mice to additional risk factors. While exposing hAβ-KI mice to LPS in the current study did not significantly increase plaque formation to the level of that seen in J20 mice, we did observe some Aβ plaques in LPS-treated hAβ-KI mice. While higher powered studies may be required to uncover such a variable effect or a later age assessed, even if LPS increased the aggregation of Aβ in our study, this did not result in any worsening of the memory or spine loss assessed in the present study suggesting that Aβ deposition is not a causal factor of spine loss in these mice. Nevertheless, given the toxic effects Aβ species are known to exert (Reiss et al., 2018), Aβ deposition may contribute to AD pathology in our mice at a later age and in addition to Aβ plaque counts, future studies should incorporate Aβ (including Aβ40 and Aβ42) analysis to better evaluate Aβ pathology. Furthermore, assessment of older hAβ-KI mice exposed to chronic inflammation is crucial and may reveal differences in AD pathologies that would support this idea.

### No changes in glial cell populations after chronic LPS treatment in hAβ-KI mice

The role of glial cell migration, proliferation, and activation in the pathology of Alzheimer's disease is well established. In our study, we corroborated our previous findings (Wright et al., 2013) that J20 mice exhibit an increase in astrocyte numbers in the hippocampus, while our hAβ-KI mice showed no change in the number of microglia or astrocytes. Previous research with hAβ-KI mice has produced conflicting results. Baglietto-Vargas et al.'s (2021) study, which aligns with our findings, demonstrated no change in the number of microglia and astrocytes, even at 18 months, as assessed by stereology. In contrast, another study employed Western blotting and densitometry analysis and observed an increase in both IBA1 and GFAP in 7 month old hAβ-KI mice (Kshirsagar et al., 2022). These contrasting results could be attributed to the different methodologies utilized across studies. Expression-based assays such as Western blotting or densitometry assess the amount of IBA1 or GFAP protein in a sample. An increase in IBA1 or GFAP protein expression (Hopperton et al., 2018), which is associated with increased microglia (Sasaki et al., 2001) and astrocyte activation (Kamphuis et al., 2012), could arise from various factors such as changes in cell numbers, cell size, or function in which more protein is generated from a single cell. In contrast, counting IBA1 and GFAP positive cell numbers using stereology quantifies the absolute cell population of microglia and astrocyte in a sample, and does not change even if the relative protein expression of IBA1 or GFAP per cell changes during microglia and astrocyte activation. Accordingly, the differences between studies using expression-based assays and those using cell counting methods suggests that IBA1 and GFAP expression and thus glial activation may increase in hAβ-KI mice without affecting the absolute glial cell population. Despite not observing any changes in glial cell numbers, Baglietto-Vargas et al. (2021) did report increases in proinflammatory cytokine expression (IL-1β and TNFα) in 18-month-old hAβ-KI mice, indicative of an increase in glial activation. Consequently, future studies aiming to examine the level of actual glial activation should incorporate cytokine analysis and/or stereology assessing activated glia, either through morphological analysis or markers of glial cell activation such as CD68, a lysosome marker expressed by microglia.

Furthermore, in our study, we found that LPS exposure in hAβ-KI mice did not lead to an increase in glial numbers. This could either suggest the limitation of our detection methods as discussed above or the absence of neuroinflammation with our LPS injection regime. As we did not observe a worsening of the AD phenotype in our LPS-injected hAβ-KI mice, we suggest that the chronic low-dose LPS regime did not trigger the intended effect. There are at least two potential explanations. Firstly, the LPS dose of 0.2 mg/kg may not have been adequate to induce a chronic inflammatory state, indicating a need for future research to explore higher doses or frequencies, despite the risk of exacerbating sickness behavior. Secondly, repeated low-dose LPS injections may have led to “immune tolerance,” wherein immune responses are suppressed upon subsequent stimulation (Biswas and Lopez-Collazo, 2009). Previous studies indicate that brain cytokine levels rise after the second exposure to low-dose LPS (0.5 mg/kg, i.p), but this increase diminishes after the fourth consecutive dose, suggesting a reduction in neuroinflammation over time (Wendeln et al., 2018). Accordingly, measuring inflammatory cytokines after multiple injections could help assess neuroinflammation changes and the development of LPS tolerance. This insight is vital for developing models of sporadic AD. If repeated LPS exposure indeed results in tolerance rather than sustained inflammation, modeling chronic inflammation as a risk factor of sporadic AD needs novel methodological approaches. Alternatively, studies could explore acute early inflammatory insults, possibly even localized in the brain through stereotaxic injections, potentially kick-starting the inflammatory cascade and thus accelerating the development of an AD phenotype.

## Conclusion

In conclusion, in the current study, we attempted to develop a more comprehensive sporadic model for AD research. Although the current LPS regime did not exacerbate any of the assessed AD pathologies, we established that hAβ-KI mice develop similar spine pathology when compared to J20 mice as well as early memory deficits at 24 weeks of age, without the development of Aβ plaques. This could be considered a significant step toward comparing different AD mouse models in an effort to model sporadic AD.

## Data availability statement

The original contributions presented in the study are included in the article/supplementary material, further inquiries can be directed to the corresponding author.

## Ethics statement

The animal study was approved by Garvan Institute and St. Vincent's Hospital Animal Ethics Committee. The study was conducted in accordance with the local legislation and institutional requirements.

## Author contributions

PR: Writing – review & editing, Writing – original draft, Visualization, Validation, Supervision, Software, Project administration, Methodology, Investigation, Formal analysis, Data curation, Conceptualization. KG: Writing – review & editing, Visualization, Validation, Methodology, Formal analysis, Data curation. AL: Validation, Methodology, Formal analysis, Writing – review & editing, Data curation. LK: Supervision, Writing – review & editing, Data curation. BV: Writing – original draft, Resources, Project administration, Funding acquisition, Conceptualization, Writing – review & editing, Supervision, Data curation.

## References

[B1] Arvanitakis Z. Wilson R. S. Bienias J. L. Evans D. A. Bennett D. A. (2004). Diabetes mellitus and risk of Alzheimer disease and decline in cognitive function. Arch. Neurol. 61, 661–666. 10.1001/archneur.61.5.661 15148141

[B2] Attar A. Liu T. Chan W. T. C. Hayes J. Nejad M. Lei K. C.. (2013). A shortened barnes maze protocol reveals memory deficits at 4-months of age in the triple-transgenic mouse model of Alzheimer's disease. PLoS ONE 8:e80355. 10.1371/journal.pone.0080355 24236177 PMC3827415

[B3] Baglietto-Vargas D. Forner S. Cai L. Martini A. C. Trujillo-Estrada L. Swarup V.. (2021). Generation of a humanized Aβ expressing mouse demonstrating aspects of Alzheimer's disease-like pathology. Nat. Commun. 12, 1–16. 10.1038/s41467-021-22624-z 33893290 PMC8065162

[B4] Bakulski K. M. Seo Y. A. Hickman R. C. Brandt D. Vadari H. S. Hu H.. (2020). Heavy metals exposure and Alzheimer's disease and related dementias. J. Alzheimers. Dis. 76:1215. 10.3233/JAD-200282 32651318 PMC7454042

[B5] Baranowski B. J. Marko D. M. Fenech R. K. Yang A. J. T. Macpherson R. E. K. (2020). Healthy brain, healthy life: a review of diet and exercise interventions to promote brain health and reduce Alzheimer's disease risk1. Appl. Physiol. Nutr. Metab. 45, 1055–1065. 10.1139/apnm-2019-0910 32717151

[B6] Barykin E. P. Mitkevich V. A. Kozin S. A. Makarov A. A. (2017). Amyloid β modification: a key to the sporadic Alzheimer's disease? *Front*. Genet. 8:261242. 10.3389/fgene.2017.00058 28555154 PMC5430028

[B7] Bekris L. M. Yu C. E. Bird T. D. Tsuang D. W. (2010). Genetics of Alzheimer disease. J. Geriatr. Psychiatry Neurol. 23:213. 10.1177/0891988710383571 21045163 PMC3044597

[B8] Beydoun M. A. Beydoun H. A. Gamaldo A. A. Teel A. Zonderman A. B. Wang Y.. (2014). Epidemiologic studies of modifiable factors associated with cognition and dementia: systematic review and meta-analysis. BMC Public Health 14, 1–33. 10.1186/1471-2458-14-643 24962204 PMC4099157

[B9] Biesmans S. Meert T. F. Bouwknecht J. A. Acton P. D. Davoodi N. De Haes P.. (2013). Systemic immune activation leads to neuroinflammation and sickness behavior in mice. Mediators Inflamm. 2013:271359. 10.1155/2013/271359 23935246 PMC3723093

[B10] Biswas S. K. Lopez-Collazo E. (2009). Endotoxin tolerance: new mechanisms, molecules and clinical significance. Trends Immunol. 30, 475–487. 10.1016/j.it.2009.07.009 19781994

[B11] Breunig J. J. Guillot-Sestier M. V. Town T. (2013). Brain injury, neuroinflammation and Alzheimer's disease. Front. Aging Neurosci. 5:52530. 10.3389/fnagi.2013.00026 23874297 PMC3708131

[B12] Brun A. Englund E. (1981). Regional pattern of degeneration in Alzheimer's disease: neuronal loss and histopathological grading. Histopathology 5, 549–564. 10.1111/j.1365-2559.1981.tb01818.x 7286917

[B13] Chakrabarti S. Khemka V. K. Banerjee A. Chatterjee G. Ganguly A. Biswas A.. (2015). Metabolic risk factors of sporadic Alzheimer's disease: implications in the pathology, pathogenesis and treatment. Aging Dis. 6:282. 10.14336/AD.2014.002 26236550 PMC4509477

[B14] Chung J. Das A. Sun X. Sobreira D. R. Leung Y. Y. Igartua C.. (2023). Genome-wide association and multi-omics studies identify MGMT as a novel risk gene for Alzheimer's disease among women. Alzheimers Dement. 19, 896–908. 10.1002/alz.12719 35770850 PMC9800643

[B15] Cortes-Canteli M. Iadecola C. (2020). Alzheimer's disease and vascular aging: JACC focus seminar. J. Am. Coll. Cardiol. 75, 942–951. 10.1016/j.jacc.2019.10.062 32130930 PMC8046164

[B16] De Wilde M. C. Overk C. R. Sijben J. W. Masliah E. (2016). Meta-analysis of synaptic pathology in Alzheimer's disease reveals selective molecular vesicular machinery vulnerability. Alzheimers Dement. 12, 633–644. 10.1016/j.jalz.2015.12.005 26776762 PMC5058345

[B17] deIpolyi A. R. Fang S. Palop J. J. Yu G. Q. Wang X. Mucke L.. (2008). Altered navigational strategy use and visuospatial deficits in hAPP transgenic mice. *Neurobiol*. Aging 29, 253–266. 10.1016/j.neurobiolaging.2006.10.021 17126954

[B18] Dorszewska J. Prendecki M. Oczkowska A. Dezor M. Kozubski W. (2016). Molecular basis of familial and sporadic Alzheimer's disease. Curr. Alzheimer Res. 13, 952–963. 10.2174/1567205013666160314150501 26971934

[B19] Fann J. R. Ribe A. R. Pedersen H. S. Fenger-Grøn M. Christensen J. Benros M. E.. (2018). Long-term risk of dementia among people with traumatic brain injury in Denmark: a population-based observational cohort study. Lancet Psychiatry 5, 424–431. 10.1016/S2215-0366(18)30065-8 29653873

[B20] Foidl B. Humpel C. (2020). Can mouse models mimic sporadic Alzheimer's disease? Neural Regen. Res. 15:401. 10.4103/1673-5374.266046 31571648 PMC6921354

[B21] Galvan V. Gorostiza O. F. Banwait S. Ataie M. Logvinova A. V. Sitaraman S.. (2006). Reversal of Alzheimer's-like pathology and behavior in human APP transgenic mice by mutation of Asp664. Proc. Natl. Acad. Sci. U. S. A. 103, 7130–7135. 10.1073/pnas.0509695103 16641106 PMC1459029

[B22] Ganesan K. Rentsch P. Langdon A. Milham L. T. Vissel B. (2024). Modeling sporadic Alzheimer's disease in mice by combining Apolipoprotein E4 risk gene with environmental risk factors. Front. Aging Neurosci. 16:1357405. 10.3389/fnagi.2024.1357405 38476659 PMC10927790

[B23] Hall A. M. Roberson E. D. (2012). Mouse models of Alzheimer's disease. Brain Res. Bull. 88, 3–12. 10.1016/j.brainresbull.2011.11.017 22142973 PMC3546481

[B24] Harold D. Abraham R. Hollingworth P. Sims R. Gerrish A. Hamshere M. L.. (2009). Genome-wide association study identifies variants at CLU and PICALM associated with Alzheimer's disease. Nat. Genet. 41, 1088–1093. 10.1038/ng.440 19734902 PMC2845877

[B25] Harris J. A. Devidze N. Halabisky B. Lo I. Thwin M. T. Yu G. Q.. (2010). Many neuronal and behavioral impairments in transgenic mouse models of Alzheimer's disease are independent of caspase cleavage of the amyloid precursor protein. J. Neurosci. 30, 372–381. 10.1523/JNEUROSCI.5341-09.2010 20053918 PMC3064502

[B26] Hodges H. (1996). Maze procedures: the radial-arm and water maze compared. Cogn. Brain Res. 3, 167–181. 10.1016/0926-6410(96)00004-3 8806020

[B27] Hong S. Beja-Glasser V. F. Nfonoyim B. M. Frouin A. Li S. Ramakrishnan S.. (2016). Complement and microglia mediate early synapse loss in Alzheimer mouse models. Science 352, 712–716. 10.1126/science.aad8373 27033548 PMC5094372

[B28] Hopperton K. E. Mohammad D. Trépanier M. O. Giuliano V. Bazinet R. P. (2018). Markers of microglia in post-mortem brain samples from patients with Alzheimer's disease: a systematic review. Mol. Psychiatry 23, 177–198. 10.1038/mp.2017.246 29230021 PMC5794890

[B29] Huang Y. Mucke L. (2012). Alzheimer mechanisms and therapeutic strategies. Cell 148:1204. 10.1016/j.cell.2012.02.040 22424230 PMC3319071

[B30] Jacobsen J. S. Wu C. C. Redwine J. M. Comery T. A. Arias R. Bowlby M.. (2006). Early-onset behavioral and synaptic deficits in a mouse model of Alzheimer's disease. Proc. Natl. Acad. Sci. U. S. A. 103, 5161–5166. 10.1073/pnas.0600948103 16549764 PMC1405622

[B31] Kamphuis W. Mamber C. Moeton M. Kooijman L. Sluijs J. A. Jansen A. H. P.. (2012). GFAP isoforms in adult mouse brain with a focus on neurogenic astrocytes and reactive astrogliosis in mouse models of Alzheimer disease. PLoS ONE 7:e0042823. 10.1371/journal.pone.0042823 22912745 PMC3418292

[B32] Karl T. Bhatia S. Cheng D. Kim W. S. Garner B. (2012). Cognitive phenotyping of amyloid precursor protein transgenic J20 mice. Behav. Brain Res. 228, 392–397. 10.1016/j.bbr.2011.12.021 22197298

[B33] Kempuraj D. Ahmed M. E. Selvakumar G. P. Thangavel R. Raikwar S. P. Zaheer S. A.. (2020). Psychological stress-induced immune response and risk of Alzheimer's disease in veterans from operation enduring freedom and operation Iraqi freedom. Clin. Ther. 42, 974–982. 10.1016/j.clinthera.2020.02.018 32184013 PMC7308186

[B34] Kitazawa M. Medeiros R. M. LaFerla F. (2012). Transgenic mouse models of Alzheimer disease: developing a better model as a tool for therapeutic interventions. Curr. Pharm. Des. 18, 1131–1147. 10.2174/138161212799315786 22288400 PMC4437619

[B35] Kozak W. Conn C. A. Kluger M. J. (1994). Lipopolysaccharide induces fever and depresses locomotor activity in unrestrained mice. Am. J. Physiol. 266, R125–R135. 10.1152/ajpregu.1994.266.1.R125 8304533

[B36] Kshirsagar S. Alvir R. V. Hindle A. Kumar S. Vijayan M. Pradeepkiran J. A.. (2022). Early cellular, molecular, morphological and behavioral changes in the humanized amyloid-beta-knock-in mouse model of late-onset Alzheimer's disease. Cells 11:733. 10.3390/cells11040733 35203382 PMC8869866

[B37] Lambert J. C. Heath S. Even G. Campion D. Sleegers K. Hiltunen M.. (2009). Genome-wide association study identifies variants at CLU and CR1 associated with Alzheimer's disease. Nat. Genet. 41, 1094–1099. 10.1038/ng.439 19734903

[B38] Lasselin J. Schedlowski M. Karshikoff B. Engler H. Lekander M. Konsman J. P.. (2020). Comparison of bacterial lipopolysaccharide-induced sickness behavior in rodents and humans: relevance for symptoms of anxiety and depression. Neurosci. Biobehav. Rev. 115, 15–24. 10.1016/j.neubiorev.2020.05.001 32433924

[B39] Leng F. Hinz R. Gentleman S. Hampshire A. Dani M. Brooks D. J.. (2022). Neuroinflammation is independently associated with brain network dysfunction in Alzheimer's disease. Mol. Psychiatry 28, 1303–1311. 10.1038/s41380-022-01878-z 36474000 PMC10005956

[B40] Lv X. Li W. Luo Y. Wang D. Zhu C. Huang Z. X.. (2013). Exploring the differences between mouse mAβ1–42 and human hAβ1–42 for Alzheimer's disease related properties and neuronal cytotoxicity. Chem. Commun. 49, 5865–5867. 10.1039/c3cc40779a 23700581

[B41] Mecca A. P. O'Dell R. S. Sharp E. S. Banks E. R. Bartlett H. H. Zhao W.. (2022). Synaptic density and cognitive performance in Alzheimer's disease: a PET imaging study with [11 C]UCB-J. Alzheimers Dement. 18, 2527–2536. 10.1002/alz.12582 35174954 PMC9381645

[B42] Mizobuchi H. Soma G. I. (2021). Low-dose lipopolysaccharide as an immune regulator for homeostasis maintenance in the central nervous system through transformation to neuroprotective microglia. Neural Regen. Res. 16:1928. 10.4103/1673-5374.308067 33642362 PMC8343302

[B43] Morris G. P. Clark I. A. Vissel B. (2018). Questions concerning the role of amyloid-β in the definition, aetiology and diagnosis of Alzheimer's disease. Acta Neuropathol. 136, 663–689. 10.1007/s00401-018-1918-8 30349969 PMC6208728

[B44] O'Leary T. P. Brown R. E. (2013). Optimization of apparatus design and behavioral measures for the assessment of visuo-spatial learning and memory of mice on the Barnes maze. Learn. Mem. 20, 85–96. 10.1101/lm.028076.112 23322557

[B45] Ownby R. L. Crocco E. Acevedo A. John V. Loewenstein D. (2006). Depression and risk for Alzheimer disease: systematic review, meta-analysis, and metaregression analysis. Arch. Gen. Psychiatry 63, 530–538. 10.1001/archpsyc.63.5.530 16651510 PMC3530614

[B46] Pozueta J. Lefort R. Ribe E. Troy C. M. Arancio O. Shelanski M. (2013). Caspase-2 is required for dendritic spine and behavioural alterations in J20 APP transgenic mice. Nat. Commun. 4:1939. 10.1038/ncomms2927 23748737 PMC4398315

[B47] Reiss A. B. Arain H. A. Stecker M. M. Siegart N. M. Kasselman L. J. (2018). Amyloid toxicity in Alzheimer's disease. Rev. Neurosci. 29, 613–627. 10.1515/revneuro-2017-0063 29447116

[B48] Sasaki Y. Ohsawa K. Kanazawa H. Kohsaka S. Imai Y. (2001). Iba1 is an actin-cross-linking protein in macrophages/microglia. Biochem. Biophys. Res. Commun. 286, 292–297. 10.1006/bbrc.2001.5388 11500035

[B49] Solfrizzi V. Panza F. Frisardi V. Seripa D. Logroscino G. Imbimbo B. P.. (2011). Diet and Alzheimer's disease risk factors or prevention: the current evidence. Expert Rev. Neurother. 11, 677–708. 10.1586/ern.11.56 21539488

[B50] Terry R. D. Masliah E. Salmon D. P. Butters N. DeTeresa R. Hill R.. (1991). Physical basis of cognitive alterations in Alzheimer's disease: synapse loss is the major correlate of cognitive impairment. Ann. Neurol. 30, 572–580. 10.1002/ana.410300410 1789684

[B51] Thakur S. Dhapola R. Sarma P. Medhi B. Reddy D. H. K. (2023). Neuroinflammation in Alzheimer's disease: current progress in molecular signaling and therapeutics. Inflammation 46, 1–17. 10.1007/s10753-022-01721-1 35986874

[B52] Vorhees C. V. Williams M. T. (2014). Assessing spatial learning and memory in rodents. ILAR J. 55, 310–332. 10.1093/ilar/ilu013 25225309 PMC4240437

[B53] Wendeln A. C. Degenhardt K. Kaurani L. Gertig M. Ulas T. Jain G.. (2018). Innate immune memory in the brain shapes neurological disease hallmarks. Nature 556, 332–338. 10.1038/s41586-018-0023-4 29643512 PMC6038912

[B54] Wightman D. P. Jansen I. E. Savage J. E. Shadrin A. A. Bahrami S. Holland D.. (2021). A genome-wide association study with 1,126,563 individuals identifies new risk loci for Alzheimer's disease. Nat. Genet. 53, 1276–1282. 10.1038/s41588-021-00921-z 34493870 PMC10243600

[B55] Wright A. L. Konen L. M. Mockett B. G. Morris G. P. Singh A. Burbano L. E.. (2023). The Q/R editing site of AMPA receptor GluA2 subunit acts as an epigenetic switch regulating dendritic spines, neurodegeneration and cognitive deficits in Alzheimer's disease. Mol. Neurodegener. 18, 1–25. 10.1186/s13024-023-00632-5 37759260 PMC10537207

[B56] Wright A. L. Zinn R. Hohensinn B. Konen L. M. Beynon S. B. Tan R. P.. (2013). Neuroinflammation and neuronal loss precede Aβ plaque deposition in the hAPP-J20 mouse model of Alzheimer's disease. PLoS ONE 8:e0059586. 10.1371/journal.pone.0059586 23560052 PMC3613362

